# Identification of required competencies for specialist nurses in infectious disease care: A modified Delphi study

**DOI:** 10.1016/j.ijnsa.2026.100573

**Published:** 2026-05-27

**Authors:** Jason P. Murphy, Johannes Haid, Emelie Härlin, Anna Hörberg

**Affiliations:** a Swedish Red Cross University, Institution for Health Sciences, Stockholm, Sweden; b Umeå University, Department of Diagnostics and Intervention, Umeå, Sweden; c Uppsala University Hospital, Uppsala, Sweden; d Dalarna University, School of Health and Welfare, Falun, Sweden

**Keywords:** Competencies, Delphi, Infectious disease, Nursing, Specialist nurse

## Abstract

**Background:**

Infectious diseases and antimicrobial resistance are increasing challenges for healthcare systems. Specialist nurses in infectious disease care are vital for prevention and management, however, formal evidence-based knowledge about the essential competencies required for this work is lacking.

**Objectives:**

To establish expert consensus on essential competencies for specialist nurses in infectious disease care in Sweden using a modified Delphi technique.

**Design:**

a three-round modified Delphi technique was utilized. Consensus was defined as ≥75% agreement.

**Participants:**

Thirty-one experts in infectious disease nursing (academic and clinical specialists) participated in three Delphi rounds.

**Setting:**

Infectious disease care settings across southern and central Sweden.

**Methods:**

A structured questionnaire comprising competency statements derived from an evidence-informed curriculum and the International Council of Nurses framework was distributed over three iterative rounds. Experts rated competencies using a 5-point Likert scale. Consensus was predefined as ≥75% agreement (ratings 4–5). Items not reaching consensus were revised and re-evaluated in subsequent rounds. Descriptive statistics were used to analyze responses.

**Results:**

Consensus was reached on 62 of 74 proposed competencies, spanning theoretical knowledge, clinical judgment, infectious disease care provision, collaboration, change management, evidence-based practice, mentoring, education, and infection control. Key competencies included advanced knowledge in microbiology, immunology, infection prevention, psychosocial health, person-centered care, and leadership during extraordinary events. Interdisciplinary collaboration and crisis management were emphasized. Competencies related to transcultural care, sustainable development, and ethical aspects did not reach consensus, highlighting areas for future research.

**Conclusion:**

The identified competencies provide a foundation for a formal framework for specialist nurses in infectious disease care, informing curricula, professional development, and integration into healthcare systems to enhance patient safety and care quality. Future research should address transcultural, leadership, and ethical dimensions within specialist nurses in infectious disease care practice.

**Registration:**

Not registered.

**Social media abstract:**

Consensus defines 62 core competencies guiding specialist infectious disease nursing practice and education.

**Reporting method:**

This study is reported in accordance with the DELPHISTAR reporting guidelines.

**Patient or public contribution:**

No Patient or Public Contribution

**What is already known**

• Infectious diseases and antimicrobial resistance challenge healthcare systems globally.
• Specialist nurses are essential for infection prevention, care, and patient safety.
• No evidence-based competency framework exists for infectious disease nursing.

Alt-text: Unlabelled box dummy alt text

**What this paper adds**

• Identifies 62 consensus-based competencies for specialist infectious disease nurses.
• Highlights key domains including clinical judgement, leadership, and infection control.
• Reveals gaps in consensus on ethical, transcultural, and sustainability competencies.

Alt-text: Unlabelled box dummy alt text

## 1 Background

Infectious diseases remain a major global public health challenge, driven by antimicrobial resistance, healthcare-associated infections emerging pathogens, and recurrent outbreaks with epidemic and pandemic potential (World Health Organization, 2024; Sun et al., 2025; European Centre for Disease Prevention and Control, 2024) . These challenges place increasing demands on healthcare systems and require advanced clinical, preventive, and organizational competencies among healthcare professionals. Nurses constitute the largest professional group in healthcare worldwide and play a central role in infection prevention, early detection, patient care, and interprofessional collaboration (The National Board of Health and Welfare, 2025). In recent decades, the complexity of infectious disease care characterized by high patient acuity, multimorbidity, and isolation requirements, has highlighted the need for nurses with advanced, specialty-specific expertise (Han et al., 2023; Wang et al., 2024).

In response to increasing infectious disease burden and the need for enhanced preparedness, Sweden has developed specialist nursing education programs within several clinical fields, including infectious disease care (Coelho, 2020). Specialist nurse education in Sweden is regulated at the national level and constitutes postgraduate education at an advanced level, building upon registered nurse qualification. Specialist nurse education in Sweden refers to postgraduate, advanced-level education for registered nurses equating to a masters’ degree. Specialist nurses are expected to possess not only advanced clinical knowledge, but also competencies in leadership, education, quality improvement, and evidence-based practice (Swedish Nursing Association, 2024; The National Association for Infection Nurses, 2024). Despite the establishment of educational programs in infectious disease care, the specific role and competency expectations of the specialist nurse in infectious disease care have not been clearly or uniformly defined. This lack of a formal, evidence-based competency framework risks role ambiguity, inconsistent educational content, and underutilization of specialist nursing competence in clinical practice. In addition, variation in curriculum content between institutions, unclear role delineation in clinical practice, and challenges in integrating specialist nursing competencies into healthcare organizations further highlight the need for a standardized competency framework.

Competency frameworks are widely used in nursing to define expectations of professional roles, guide curriculum development, support quality assurance, and strengthen patient safety (European Centre for Disease Prevention and Control, 2022). The International Council of Nurses (ICN) defines competence as a dynamic combination of knowledge, skills, attitudes, values, and professional judgement that enables effective performance in a specific context (ICN, 2009). In this study, the different dimensions of competence, including personal attributes and professional values, were integrated into the development of competencies by being operationalized as observable and assessable behaviors within specific clinical, educational, and organizational contexts. In line with this definition, competence in this study is understood as encompassing cognitive, technical, psychomotor, and interpersonal skills, as well as personal attributes such as ethical awareness, critical thinking, and clinical judgement. This conceptualization acknowledges that specialist nursing competence extends beyond task-based skills and includes the capacity to integrate knowledge into complex clinical and organizational situations.

While competency frameworks exist for several specialist nursing roles both nationally and internationally (Swedish Nursing Association, 2024), there is currently no empirically grounded framework that specifically delineates the competencies required for specialist nurses in infectious disease care. Existing descriptions often vary in scope, methodological quality, and contextual relevance, limiting their utility for education and workforce development (Batt et al., 2020). Internationally, Delphi-based studies and expert frameworks have been used to define specialist nursing competencies in areas such as infection prevention, public health, and advanced clinical care, providing a methodological foundation for consensus-based competency development (Keeney, 2011).

Delphi-based studies and competency development research have demonstrated the value of structured, multidimensional frameworks, often comprising domains such as professional development, infection control, clinical care, and emergency preparedness (Wu et al., 2024). Recent studies have described both competency-based training systems and validated assessment instruments encompassing core domains such as professional development, infection prevention, clinical care, humanistic competence, and emergency response (Wu et al., 2024; Wu et al., 2021). In addition, qualitative research exploring clinical and academic perspectives highlights the ongoing need for structured curricula and clearly defined specialist roles, identifying gaps in educational preparation, variability in training content, and challenges in implementing specialist competencies in practice (Gorjian et al., 2024). These findings indicate that although international research has advanced the conceptualization and structuring of specialist nursing competencies, substantial variation remains across educational and healthcare systems. This underscores the need for standardized, contextually relevant, and evidence-based competency frameworks.

## 2 The study

This study aims to achieve expert consensus on vital competencies required for specialist nurses in infectious disease care, using a modified Delphi technique with a quantitative approach.

## 3 Methods

### 3.1 Design

A modified Delphi technique with a quantitative approach was utilized. The Delphi technique assumes that the opinion of an expert group is more valid than that of an individual (Keeney, 2011). The Delphi technique is a common consensus method often used to explore emerging fields of research with limited empirical evidence (Keeney, 2011).

### 3.2 Study setting

This study was conducted within the Swedish infectious disease care setting. As of 2024, Sweden has approximately 90,000 registered nurses, including approximately 4000 specialist nurses distributed among the eleven specialist categories, a 5.9% increase since 2020 (Swedish Association of Local Authorities and Regions, 2025).

### 3.3 Expert panel

Guided by the study aim, a purposive snowball sampling strategy was employed to recruit an expert panel. In this study, an expert was defined as a registered nurse with specialist competence in infectious disease care, achieved through formal postgraduate education, extensive clinical experience, or a combination of both (Keeney et al., 2006; Petty, 2015).

Two expert categories were included: academic experts and clinical experts. Inclusion criteria: Academic experts were required to be registered nurses holding at least a master’s degree in infectious disease care. Clinical experts were defined according to Benner’s model as registered nurses with a minimum of five years of clinical experience in infectious disease care (Benner, 1984). Exclusion criteria were insufficient experience or lack of relevant expertise.

To preserve participant confidentiality and maintain quasi-anonymity, detailed institutional affiliations were not collected. However, the expert panel was recruited across professional networks in infectious disease care, and participants are considered to primarily represent healthcare and academic settings in the middle and southern regions of Sweden.

A total of 57 formal email invitations were distributed. Initial participants were identified through the authors’ professional networks and subsequently invited to nominate additional eligible experts. Invitation emails included detailed information about the study design, confidentiality, and estimated time commitment. Of the 34 experts who agreed to participate, 31 completed the first-round questionnaire and provided informed consent, thereby constituting the final expert panel. Participants were drawn from seven regions across the middle and southern parts of Sweden, encompassing both urban and rural settings and reflecting variation in population density, healthcare organization, and infectious disease care delivery contexts.

The expert panel had a mean of 15.3 years (± 9.77) of experience in infectious disease care and a mean age of 44.1 years (± 10.1) (Table 1). Fourteen participants were classified as academic experts, defined as registered nurses holding a master’s degree or higher in infectious disease care, indicating advanced theoretical knowledge and specialist competence within the field (Extension School, 2022). The remaining seventeen participants constituted the clinical expert group and met Benner’s criteria for expertise, defined as a minimum of five years of clinical experience in infectious disease care (Benner, 1984). Table 2

**Table 1:** Demography of experts in the study panel.

Experts (n = 31)	Age (years)	Years of experience (years)
**Mean**	44.1	15.3
**Median**	42	12
**Minimum**	28	5
**Maximum**	67	46

**Table 2:** Expert panel response rate per round.

	Academic experts (n)	Clinical experts (n)	Total (n/total previous round)
Round 1	14	17	31/34 (91%)
Round 2	13	14	27/31 (87%)
Round 3	11	14	25/27 (92%)
Overall response rate			27/34 (79%)

### 3.4 Data collection

The modified Delphi technique is an iterative process involving multiple rounds in which items are distributed to a panel of experts until consensus is reached (Keeney, 2011).

### 3.5 Questionnaire development and content validity

The initial questionnaire was informed by the specialist nursing curriculum in infectious disease care at the Swedish Red Cross University and the International Council of Nurses. The first author was part of the academic team responsible for designing the curriculum, which was constructed through a structured review of current international and national literature on infectious disease nursing, competency-based education, and specialist nursing roles, alongside benchmarking against comparable international master’s-level programs in infectious and communicable disease nursing.

The curriculum development process integrated evidence from international competency frameworks (including those published by professional nursing and public health organizations), peer-reviewed research on specialist nurse competencies, and learning outcomes from established academic programs in Europe and beyond (ICN, 2009; Swedish Red Cross University, 2025). This process ensured that the curriculum reflected contemporary scientific knowledge, clinical practice requirements, and internationally recognized expectations for advanced nursing practice within infectious disease care.

The development of the initial competency items was guided by the International Council of Nurses’ conceptual framework of competence (ICN, 2009). In line with this framework, competencies were formulated to reflect an integrated combination of knowledge, skills, professional judgement, and underlying values, with personal attributes operationalized through observable competency statements.

The questionnaire items used in the present Delphi study were derived from this evidence-informed curriculum and operationalized as discrete competency statements suitable for expert evaluation. The questionnaire content thus was grounded in a broader synthesis of existing research and international educational standards.

To further strengthen content clarity and relevance, the draft questionnaire underwent pilot testing with six specialist nursing students in infectious disease care, resulting in minor revisions to wording and structure prior to data collection. Following round one of the Delphi process, experts were also invited to propose additional competencies, allowing the instrument to be refined iteratively based on expert knowledge and clinical experience beyond the original curriculum-derived items (Keeney, 2011).

The first section of the questionnaire included informed consent and demographic items, while the second section comprised 54 proposed specialist nurses in infectious disease care competencies (Appendix 2).

Competency categories were specified in advance and guided by the International Council of Nurses specialist nurse competency framework and the aforementioned curriculum (ICN, 2009). The competency categories (e.g., theoretical knowledge, treatment of infectious disease, and clinical judgement) were predefined and guided by the International Council of Nurses’ competency framework and the evidence-informed curriculum. These categories provided a structural framework for organizing the initial competency items rather than emerging inductively from the Delphi process. The subsequent Delphi rounds were used to evaluate, refine, and reach consensus on the relevance and importance of competencies within these predefined domains.

### 3.6 Delphi rounds

Experts were asked to rate the importance of each item is using a 5-point Likert scale: 5=Very important, 4=Quite important, 3=Neither important nor unimportant, 2 Quite unimportant, 1=Not at all important. Experts were invited to suggest additional competencies beyond those initially proposed by the authors. In Delphi studies, providing experts with summary statistics for each item constitutes group feedback and enables informed reconsideration of individual judgments. Thus, after rounds 1 and 2, each expert received feedback that included their individual ratings for each statement alongside the group’s mean value for each statement, allowing them to reconsider and adjust their assessments. This feedback informed item retention or exclusion based on the predefined consensus criterion rather than written qualitative comments. The revised questionnaire, containing items that had not yet reached consensus along with newly added items was subsequently distributed to the experts in rounds 2 and 3 for further rating.

Three Delphi rounds were considered sufficient to achieve consensus while minimizing the risk of participant fatigue (Keeney et al., 2006).

The questionnaire was distributed via Google Forms and email, with follow-up reminders issued to enhance response rates.

### 3.7 Data analysis

The consensus level for this study was predefined at 75% (Keeney, 2011). The results of responses were trichotomized in ratings of 4–5, 3 and 2–1 (Jirwe et al., 2009). Competencies with rating scores of 4–5 were deemed important, ratings 1–2 non-important and rating scores of 3 neither important nor non-important (Furtado et al., 2024). Data were analyzed with descriptive statistics using Jamovi version 2.6 for measuring means and standard deviation (The jamovi project, 2024). Means and standard deviations were used to provide feedback with the mean representing group opinion while standard deviation indicated consensus with lower standard deviations indicating higher consensus (Greatorex and Dexter, 2000). After each Delphi round, items achieving ≥75% agreement (ratings of 4–5 on the 5-point Likert scale) were considered to have reached consensus and were removed from subsequent rounds, while items below the threshold were retained, revised if necessary, and re-evaluated in the next round.

### 3.8 Ethical considerations

All experts received written information concerning the study. All participants received study information and gave informed consent (Appendix 1). The Delphi process ensured quasi-anonymity. Quasi-anonymity in this study refers to participants being aware of the identities of other experts in the panel, but individual responses were not disclosed, ensuring that responses remained anonymous within the Delphi process. Participation was voluntary and confidential (Keeney et al., 2006). No sensitive data were collected, and the study was approved by the Swedish Regional Ethical Review Board (dnr 2024–08,196-01).

## 4 Results

**Fig. 1:**
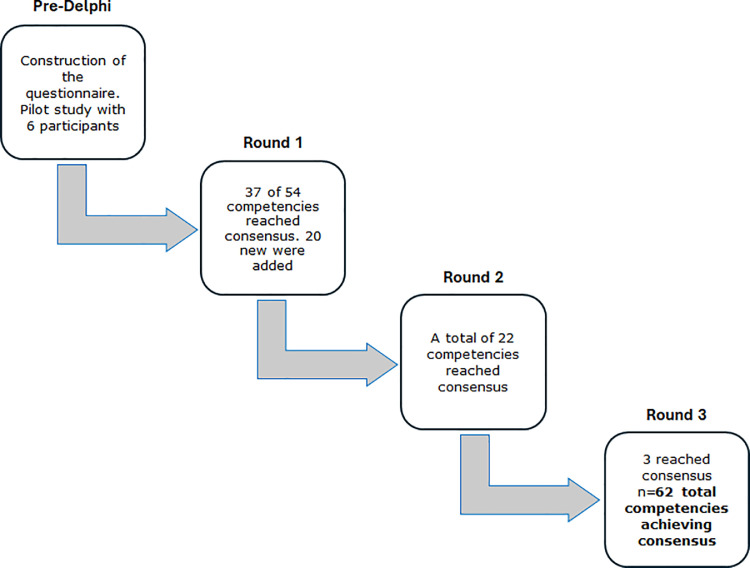
Delphi flow chart.

The aim of this study was to achieve expert consensus on the vital competencies required for specialist nurses in infectious disease care, using a modified Delphi technique with a quantitative approach. The expert panel in this study reached consensus on 62 competencies out of the 74 described (Fig. 1). The overall response rate was 79% (25/34).

The Delphi process is presented stepwise across three rounds, illustrating the iterative refinement of competencies through expert consensus. The progression of items across rounds, including their mean ratings, standard deviations, and consensus outcomes, is detailed in Table 3, Table 4. Items reaching the predefined consensus threshold (≥75%) were retained and removed from subsequent rounds, while remaining items were carried forward for re-evaluation. Additional competencies proposed by experts were incorporated following review and included in subsequent rounds.

**Table 3:** Competencies that reached consensus (75%).

	Competency domains	* Competencies added in by experts in round two	Mean	SD	Round competency reached consensus
*1*	*Theoretical knowledge*				
1	In-depth knowledge of the most common infectious diseases concerning etiology, physiology, pathophysiology, and treatment		4.74	0.45	1
2	In-depth knowledge in microbiology		4.03	0.66	1
3	In-depth knowledge in immunology		4.00	0.82	1
4	In-depth knowledge of vaccines and vaccinations*		4,04	0,65	2*
5	Basic knowledge of the Swedish vaccination program		4.12	0.60	3
*2*	*Treatment of infectious disease*				
6	Advanced medical-technical skills related to the care and treatment of infectious diseases*		4,44	0,58	2*
7	In-depth knowledge of various types of vascular access devices and associated complications, to minimize the risk of healthcare-related injuries		4.58	0.62	1
8	Advanced knowledge of, and the ability to stay current with, microbiological sampling and specimen handling		4.26	0.77	1
9	Knowledge and understanding of drug interactions, as well as complications and adverse effects associated with pharmacological treatment of infectious diseases*		4,07	0,62	2*
10	Ability to assess the need for, propose, and justify the initiation of central venous access		4.16	0.74	1
11	Ability to critically review and evaluate medication prescriptions in the management of infectious diseases		4.15	0.72	2
12	Knowledge and understanding of empirical antibiotic selection for adult patients in hospital settings		3.85	0.60	2
*3*	*Infectious care provision*				
13	In-depth knowledge of, and the ability to actively promote, psychosocial health among individuals living with or diagnosed with chronic infectious diseases		4.48	0.57	1
14	Ability to identify and contextualize the determinants of health for vulnerable and marginalized groups within infectious care, as well as to plan, design, and evaluate appropriate nursing interventions		4.32	0.79	1
15	Enhanced understanding of, and the ability to justify, person-centered activities and processes within infectious care		4.52	0.63	1
16	Advanced knowledge in assessing and analyzing nursing needs, as well as in justifying nursing interventions related to isolation care		4.87	0.34	1
17	Advanced knowledge in assessing and analyzing nursing problems, as well as in justifying nursing interventions for infectious diseases		4.71	0.59	1
18	In-depth knowledge of preventive nursing interventions for patients with primary and secondary immunodeficiency		4,07	0,68	2
19	In-depth knowledge of preventive nursing interventions for patients with pneumonia		4.74	0.51	1
20	In-depth knowledge of preventive nursing interventions for patients with central nervous system infections		4.74	0.45	1
21	In-depth knowledge of preventive nursing interventions for patients with spondylodiscitis		4.65	0.55	1
22	In-depth knowledge of preventive nursing interventions for patients with endocarditis		4.74	0.51	1
23	In-depth knowledge of preventive nursing interventions for patients with tuberculosis		4.71	0.53	1
24	In-depth knowledge of preventive nursing interventions for patients undergoing long-term antibiotic treatment		4.65	0.55	1
25	Advanced knowledge and understanding of healthcare-associated infections concerning their onset, preventive measures, care, and treatment*		4.78	0.42	2*
*4*	*Clinical judgement*				
26	Good clinical situational awareness to prevent critical situations for patients		4.55	0.62	1
27	Ability to promptly detect, assess, and analyze symptoms of necrotizing fasciitis		4.71	0.53	1
28	Ability to promptly detect, assess, and analyze symptoms of acute bacterial meningitis		4.74	0.51	1
29	Ability to promptly detect, assess, and analyze symptoms of septic chock		4.94	0.36	1
30	Ability to systematically conduct and evaluate physical health assessments with an ethical and equitable approach		4,15	0.72	2
31	Ability to systematically conduct and evaluate mental health assessments with an ethical and equitable approach.		4.07	0.66	2
*5*	*Collaboration and interprofessional relationships*				
*32*	Knowledge and understanding of leadership, planning, and interprofessional collaboration in the provision of nursing care for infectious diseases in the context of war, disasters, and humanitarian interventions*		4.00	0.70	3*
*33*	Ability to lead interprofessional teams during extraordinary events*		4.08	0.76	3*
34	Competence for interprofessional collaboration with other professional groups, authorities, and organizations*		4.15	0,86	2*
*5*	*Change management*				
35	Ability to contribute to the reduction of antibiotic resistance through advanced knowledge of antibiotics		4.52	0.77	1
36	Advanced knowledge and understanding of the planning, implementation, and evaluation of quality improvement initiatives*		4.15	0.72	2*
6	*Evidence-based practice*				
37	Ability and proficiency to search for, evaluate, and implement nursing research and theories of care		4.23	0.67	1
38	The ability to promote an evidence-based knowledge culture in the workplace		4.58	0.56	1
	*Research*				
39	Ability to conduct systematic literature reviews and apply evidence-based guidelines within the profession-specific organization		4.06	0.86	1
40	Ability to initiate and participate in research and development activities based on a scientific approach		4.26	0.73	1
*7*	*Mentoring and coaching*				
41	Ability to coach colleagues in enhancing situational awareness to prevent critical incidents for patients		4.48	0.68	1
42	Advanced pedagogical knowledge and the ability to support less experienced colleagues through reflection and bedside supervision*		4,56	0,70	2*
8	*Education and teaching*				
43	Knowledge of, and pedagogical skills to educate patients in self-care, pathology, and transmission routes related to infectious diseases		4,48	0,70	2*
44	Advanced pedagogical knowledge and the ability to take responsibility for supervising undergraduate students		3,96	0,77	2
45	Advanced pedagogical knowledge and the ability to take responsibility for the supervision of students at the advanced level		4.13	0.76	1
46	Advanced pedagogical knowledge and the ability to contribute to education within the clinical setting		4.42	0.67	1
47	Advanced pedagogical knowledge and the ability to participate in external educational initiatives		3.96	0.81	1
48	Advanced pedagogical knowledge and the ability to foster experiential learning among nursing students		4.30	0.67	2
*7*	*Infection control and prevention*				
49	Advanced knowledge of multidrug-resistant bacteria concerning their emergence, modes of transmission, and strategies for interrupting transmission pathways*		4.74	0.45	2*
50	Advanced knowledge of bloodborne infections regarding their origin, transmission routes, and methods for disrupting these transmission pathways		4.74	0.45	2*
51	Advanced knowledge of the care and treatment of patients with multidrug-resistant bacteria		4.67	0.56	2*
52	Knowledge and understanding of transmission pathways in hospital settings, as well as the ability to conduct risk assessments for infection following exposure		4.70	0.54	2*
53	Knowledge and understanding of transmission pathways in the community, along with the ability to perform risk assessments for infection following exposure		4.56	0.58	2*
54	Knowledge and understanding of epidemiological transmission of infectious diseases in the context of war, conflict, and disasters		3.96	0.70	2*
55	Advanced knowledge and understanding of the donning and doffing of appropriate personal protective equipment for various infectious diseases		4.77	0.50	1
56	Ability to assess, propose, and justify infection prevention measures		4.81	0.40	1
57	Fundamental knowledge of the Swedish Communicable Diseases Act		4.58	0.56	1
58	Basic knowledge of infection control measures for highly contagious and serious diseases		4.74	0.45	1
59	Knowledge and understanding of guidelines for working with highly contagious and serious diseases		4.68	0.60	1
60	Fundamental knowledge of the symptoms associated with highly contagious and serious diseases		4.68	0.48	1
61	Epidemiological knowledge of infectious diseases at the national level		4.06	0.85	1
62	Epidemiological knowledge of infectious diseases at a global level		4.22	0.50	2

**Table 4:** Competencies that did not reach consensus (75%).

	Competency domains	* Competencies added in by experts in round two	Consensus level	Mean	SD
1	In-depth knowledge of neglected tropical infectious diseases regarding their etiology, physiology, pathophysiology, and treatment		64% ≥4	3.68	0.90
2	Knowledge of the care and management of patients exposed to violence, and fundamental understanding of the association between exposure to violence and health		52% =3	2.96	0.79
3	Enhanced understanding of sustainable development with consideration of ethical aspects, gender equality, and human rights		56% ≥3	3.56	0.77
4	Advanced knowledge and the ability to promote and justify sustainable decision making within the organization		44% =3	3.36	0.90
5	Advanced knowledge of, and the ability to promote and justify, sustainable decision making within the organization’s operations*		44% ≥4	3.60	0.87
6	Advanced knowledge of healthcare and nursing from a transcultural perspective		72% ≥4	3.80	0.76
7	Ability to propose and justify pharmacological treatments related to infectious diseases		72% ≥4	3.65	0.64
8	Knowledge and understanding of empirical antibiotic selection for adults in outpatient care		56% ≥4	3.52	0.71
9	Advanced knowledge of risk prevention strategies within the organization		72% ≥4	3.84	0.85
10	Knowledge and proficiency in managing incident reporting within the organization through investigation, analysis, and feedback to colleagues		48% ≥4	3.32	0.95
11	In-depth knowledge and understanding of risk prevention efforts within the organization*		52% ≥4	3.60	0.87
12	Evaluate health-promoting and disease-preventive measures from ethical, societal, and scientific perspectives, with consideration of human rights		60% ≥4	3.68	0.75

### 4.1 Round one

The initial questionnaire comprised 54 competencies derived from the evidence-informed curriculum. In this round, 37 competencies (68%) reached the predefined consensus level (≥75%) and were therefore retained as final competencies and removed from further evaluation (Table 3).

The remaining 17 competencies did not reach consensus and were carried forward to the next round for re-evaluation.

In addition to rating the initial items, experts proposed 20 new competencies, which were reviewed by the research team to ensure alignment with the study aim and conceptual framework. These newly identified competencies were incorporated into the subsequent round alongside the retained non-consensus items.

As a result, a total of 37 competencies were included in Round 2 for further evaluation.

Expert-suggested competencies are indicated in the tables (*), and their progression across Delphi rounds reflects iterative refinement based on expert consensus.

### 4.2 Round two

A total of 37 competencies (including both non-consensus items from Round 1 and newly added expert-suggested competencies) were evaluated in round 2.

In this round, an additional 22 competencies reached the consensus threshold, resulting in a total of 59 competencies achieving consensus across rounds 1 and 2 (79% n = 59).. These items were subsequently removed from further evaluation.

Fifteen competencies did not reach consensus and were retained for further reconsideration in round 3. No new competencies were introduced in this round.

Consequently, fifteen competencies were retained and forwarded to the expert panel in Round 3. A comprehensive overview of all expert-suggested competencies, their mean ratings, agreement levels, and outcomes across rounds is presented in Table 3, Table 4.

### 4.3 Round three

In Round 3, the expert panel reached consensus on three more competencies resulting in consensus on 62 vital competencies out of the total 74 with a mean ranging from 3.96 to 4.94 (SD 0.34–0.86) (Table 3). Twelve competencies did not reach the predetermined consensus level of 75% (Table 4).

## 5 Discussion

This study was grounded in the International Council of Nurses’ conceptualization of specialist nursing competence as an integrated combination of knowledge, skills, professional judgement, and personal attributes applied within complex clinical contexts (ICN, 2009). Interpreted through this theoretical lens, the competencies identified by the expert panel reflect a multidimensional construct encompassing clinical, relational, educational, and organizational dimensions of specialist nursing practice.

The findings of this study align with and extend international research on competency frameworks for specialist nurses in infectious disease care. Consistent with previous studies, the identified competencies reflect a multidimensional structure that includes clinical expertise, infection prevention and control, education, leadership, and interprofessional collaboration. Similar domain structures have been described in competency-based training systems and validated instruments for infectious disease specialist nurses, which highlight core areas such as clinical care, infection control, professional development, and emergency preparedness (Wu et al., 2024; Wu et al., 2021).

The emphasis on clinical knowledge, diagnostic reasoning, and infection prevention is also consistent with international curriculum-based frameworks for advanced practitioners in infectious diseases, which underscore the importance of microbiological competence, antimicrobial management, and patient-centered care (Smith et al., 2024). Together, these findings support a shared international understanding of specialist nursing competence as a complex and integrated construct that extends beyond technical skills to include cognitive and relational capabilities.

However, some differences were observed. While international competency frameworks frequently include domains such as professionalism, ethical practice, and culturally sensitive care as core components (ICN, 2009; Smith et al., 2024), these areas did not reach consensus in the present study. Similar variability has been reported in qualitative research on infection prevention and control nursing, which identified inconsistencies in curriculum content, role expectations, and implementation across settings (Gorjian et al., 2024). These discrepancies may reflect contextual differences in healthcare systems, educational structures, and clinical priorities. In the Swedish setting, a stronger emphasis may be placed on clinical and operational competencies directly related to patient care, potentially influencing expert prioritization.

The findings also highlight the importance of context-specific competencies, particularly those related to leadership and interprofessional collaboration during extraordinary events such as pandemics, disasters, and humanitarian crises. While these competencies are present in previous research, they are often less explicitly emphasized, suggesting that the present study contributes a more practice-oriented and contextually grounded specification of specialist nursing competence in infectious disease care (Wu et al., 2024).

These findings demonstrate both convergence and variation in specialist nursing competencies across international contexts. While core domains appear consistent, differences in emphasis underscore the need for competency frameworks that are both evidence-based and adaptable to local healthcare contexts.

Furthermore, the findings further emphasize the central role of clinical judgement in specialist nursing practice. The expert panel reached consensus on all competencies related to clinical assessment, highlighting the importance of systematic and ethically grounded patient evaluation. This aligns with previous research demonstrating that clinical and technical competence are central to specialist nursing roles in Europe (Dury, 2014), and underscores the importance of integrating knowledge and decision-making in complex clinical environments.

Similarly, competencies related to infectious disease care provision reflected a strong focus on both clinical and preventive aspects of care. For example, consensus was achieved regarding competencies related to tuberculosis management and chronic infectious diseases. Previous research has identified the lack of specialist nurses in tuberculosis care as a barrier to effective infection control (Collin et al., 2018), highlighting the relevance of these findings. The inclusion of competencies related to psychosocial health and determinants of health further reflects the growing recognition that effective infectious disease care extends beyond biomedical management, particularly in conditions such as HIV and hepatitis, where stigma, adherence, and comorbidities play significant roles (Vasylyev et al., 2024; Takeda et al., 2019).

The lack of consensus regarding ethical competencies represents a notable result. Ethical and legal practice is widely recognized as a core component of specialist nursing competence (ICN, 2009), with previous studies emphasizing the importance of ethical decision-making, particularly during infectious disease outbreaks (Zumstein-Shaha and Grace, 2023). The lack of consensus in this study may reflect variability in how ethical competence is conceptualized or operationalized in practice and suggests an important area for future research and educational development.

Leadership and interprofessional collaboration were identified as critical competencies, particularly in the context of crises. The ability to lead interprofessional teams and coordinate care during extraordinary events such as pandemics, disasters, and humanitarian situations reflects lessons learned from recent global health challenges. Previous research has demonstrated that such contexts require coordinated multidisciplinary responses to manage infectious disease outbreaks effectively (Marou et al., 2024). These findings reinforce the importance of preparing specialist nurses for leadership roles in complex and rapidly evolving healthcare environments.

Pedagogical competencies were also prominent, with several competencies related to teaching, supervision, and mentoring reaching consensus. This aligns with previous studies identifying education and mentorship as integral components of specialist nursing roles (Dury et al., 2014; Wu et al., 2021). The ability to support less experienced colleagues and contribute to knowledge dissemination is particularly important in infectious disease care settings characterized by high complexity and rapid knowledge development. Moreover, mentorship has been associated with improved staff retention and professional development (Hörberg et al., 2017), further reinforcing its importance within specialist nursing practice.

These findings may have implications for the development and revision of specialist nurse education in infectious disease care in Sweden. The identified competencies provide a structured, evidence-based foundation that can inform curriculum design, ensuring alignment between educational content and clinical practice requirements. In particular, the emphasis on clinical judgement, infection prevention and control, leadership, and interprofessional collaboration suggests that these areas should be prioritized and further strengthened within existing programs. At the same time, the lack of consensus regarding ethical, transcultural, and sustainability-related competencies highlights areas that may require clearer definition and integration into educational curricula. Incorporating these competencies into formal education may support more comprehensive preparation of specialist nurses, enhance role clarity, and facilitate more effective utilization of specialist competence within healthcare systems.

Overall, these findings reinforce the International Council of Nurses’ conceptualization of specialist nursing competence as a dynamic and multidimensional construct while providing a context-specific elaboration relevant to infectious disease care. This study contributes to the international literature by offering a consensus-based competency framework that both reflects and refines existing models and highlights the importance of adapting competency frameworks to specific healthcare contexts.

### 5.1 Method discussion

As the sample and consensus process in each Delphi technique study is unique, there are no clear guidelines on how validity and reliability should be established in Delphi technique studies (Keeney et al., 2006).By consulting experts, valuable insights are generated which contribute to answering the research question. Due to the non-random nature of the sampling process, there is an inherent risk of selection bias, as individuals with a greater interest in the topic are more likely to participate across multiple rounds.

To mitigate framing bias, Delphi panels should be composed of heterogeneous experts (Keeney et al., 2006). This study included clinical experts with ≥5 years of experience and academic experts holding at least a master’s degree in infectious disease care. While the inclusion of nurse educators and managers could have broadened perspectives and enhanced validity (Furtado et al., 2024) they were not targeted for recruitment. The credibility of Delphi findings depends on diverse representation throughout the process and selective dropout may compromise panel representativeness (Engels and Kennedy, 2007).

The validity of study results in Delphi technique studies is positively affected by the iterative rounds since this allows the experts continuous reflection. However, this iterative process can lead to participant fatigue lowering response rates which in turn can compromise consecutive rounds and reduce representation of various perspectives and opinions. Indeed, a significant risk to validity of a Delphi study is participant attrition (Keeney, 2011). This study was limited to three Delphi rounds to reduce participant fatigue. Response rates were high: 91% (Round 1), 87% (Round 2), and 92% (Round 3), exceeding the recommended 70% threshold for rigor. Round durations were adjusted for holidays, with the second round extended to 12 days. A 14-day response window is suggested as reasonable. To minimize dropout, quasi-anonymity was used to encourage participation, and the panel size was kept large to absorb attrition.

Confirmability and dependability in Delphi studies rely on clear consensus criteria (Shang, 2023) . This study set a 75% threshold, leaving three competencies just below at 72%. While no standard exists, a predefined cut-off is good practice, and 75% is commonly accepted with studies recommending 70–80% unless addressing sensitive topics (Shang, 2023). Feedback was quantitative, showing mean ratings and agreement percentages. A stricter threshold might have improved accuracy at the risk of too few competencies reaching consensus, potentially omitting relevant competencies despite broad expert support.

The competency on transcultural perspective narrowly missed consensus, potentially due to experts’ varied pre-understanding, as no definition was provided. A revised phrasing such as “knowledge of cultural nursing” might have improved clarity. The initial questionnaire, authored by the researchers, was triangulated with the researchers to reduce bias and piloted with six experts for clarity. Despite these efforts, some competencies were flagged as ambiguous, such as “good clinical situational awareness,” which is subjective and may not align with the international council of nurses’ competency definition (ICN, 2009). This competency nonetheless reached consensus. Conversely, a broadly formulated competency on evaluating health measures from ethical, societal, and scientific perspectives did not. More extensive pilot testing and alternative questionnaire development approaches could have enhanced clarity and reduced bias. In addition, a clearer link between the competency items and their theoretical basis may have influenced the level of agreement among experts, as different interpretations of more complex concepts could have affected consensus.

To increase credibility, additional research methods could have been used pre- Delphi and in Round 1. It has been argued that the first round of a traditional Delphi technique study could be particularly important to stimulate commitment and a way to obtain qualitative information from experts on the research question (Engels and Kennedy, 2007). A more traditional approach to the Delphi technique employs interviews in the first round to generate ideas for the questionnaire in the subsequent rounds (Keeney, 2011).

A further limitation is that the study focused on the perceived importance of competencies and did not explicitly assess their contextual relevance, practical applicability, or feasibility of implementation in clinical practice. Although the Delphi technique is well suited for establishing consensus on key competencies, it does not capture how these competencies can be operationalized within diverse healthcare settings.

## 6 Conclusion

The importance of the specialist nurse in infectious disease care has become clearer in the light of recent infectious disease epidemics and pandemics. The complexity of infectious disease care is evident with increasing rates of hospital acquired infections, antimicrobial resistance, and care of isolated and comorbid patients.

In conclusion, the competencies that reached consensus among the expert group suggest that these competencies may provide initial guidance on the contents of a competency framework for the Swedish specialist nurse in infectious disease care. These competencies may also provide insight into the professional role of the specialist nurse in infectious disease care and enhance the understanding of competencies required to provide quality care to infectious patients in the study setting.

Future research should explore transcultural knowledge and leadership within the specialist nurses in infectious disease care role, as well as core competencies such as person-centered care, collaboration, evidence-based practice, and informatics. In addition, investigating ethical aspects such as patient autonomy, confidentiality, and equitable access to care is essential for ensuring that the development and implementation of competencies are aligned with ethical standards and support person-centered, and sustainable healthcare practices.

Furthermore, future research should examine the implementation, feasibility, and real-world applicability of the identified competencies across different organizational and clinical contexts.

## Consent for publication

Not applicable.

## Availability of data and materials

To protect participant privacy, the data is not publicly available. Data supporting the study’s findings are available from the corresponding author upon request.

## Clinical trial number

not applicable

## Declaration of generative AI and AI-assisted technologies in the manuscript preparation process

During the preparation of this manuscript, the authors used Copilot to assist with grammar, spelling, and structural suggestions. No AI tools were used for data analysis, interpretation, or generation of scientific content

## CRediT authorship contribution statement

**Jason P. Murphy:** Writing – review & editing, Writing – original draft, Validation, Methodology, Formal analysis, Conceptualization. **Johannes Haid:** Writing – review & editing, Writing – original draft, Validation, Formal analysis, Data curation. **Emelie Härlin:** Writing – review & editing, Conceptualization. **Anna Hörberg:** Writing – review & editing, Validation, Methodology.

## Declaration of competing interest

The authors declare that they have no competing interests.

## Appendix Supplementary materials

Image, application 1

Image, application 2
