# Supplementary material for: Identification of required competencies for specialist nurses in infectious disease care: A modified Delphi study
Source: Int J Nurs Stud Adv. 2026 May 27;11:100573. doi: 10.1016/j.ijnsa.2026.100573 (PMC13266131; doi:10.1016/j.ijnsa.2026.100573)
Supplement: Supplementary file 1 [file mmc1.docx]

**Information for Research Participants**

I would like to invite you to participate in a research project. This document provides information about the project and what participation entails.

What is the project about and why am I being asked to participate?

I am a specialist nurse in infectious diseases nursing and active within the Swedish Association of Infectious Disease Nurses (Riksföreningen för infektionssjuksköterskor, RFI). I intend to conduct a study to support the work undertaken by RFI to publish a competency framework for specialist nurses in infectious diseases nursing.

The aim of the study is to identify key competencies for specialist nurses specializing in infectious diseases nursing.

You are invited to participate because I consider you to be an expert in nursing care within the field of infectious diseases.

**What will happen if I choose to participate?**

The study will be conducted using the Delphi method. As a participant, you will be asked to complete a questionnaire on three occasions, referred to as rounds. The questionnaire consists of six demographic questions and 54 competency-related items in which you will rate the importance of each competency using a five-point Likert scale.

The time required to complete the questionnaires will vary between rounds, but each questionnaire is estimated to take approximately 15–25 minutes to complete. There are no right or wrong answers to the questionnaire items. The study seeks your informed and professional opinion.

After each round, you will receive a summary of all participants’ responses. The goal is to achieve consensus regarding the core competencies required of specialist nurses in infectious diseases care. A consensus level of 75 percent will be applied, meaning that competencies for which 75 percent of participants provide the same rating will not be included in the questionnaire for the following round.

**What will happen to my personal data?**

The project will collect and process information about you. As a participant, you will be informed of the names of the other participants in the study. Your responses in the questionnaire study will be treated confidentially and presented in a form in which only the authors will be able to identify individual participants’ responses.

Before the questionnaires in rounds two and three, you will receive a brief written summary of the panel’s mean responses together with your own previous response.

The Swedish Red Cross University is responsible for the processing of your personal data. In accordance with the EU General Data Protection Regulation (GDPR), you have the right to access, free of charge, the personal data processed about you within the project and, if necessary, to have any inaccuracies corrected. You also have the right to request deletion of your data and restriction of the processing of your personal data. However, the rights to deletion and restriction do not apply when the data are necessary for the current research project.

If you wish to access your data, please contact:

Jason Murphy
Swedish Red Cross University
Box 1059, 141 21 Huddinge, Sweden
Hälsovägen 11C
Telephone: +46 (0)8-587 516 00
Mobile: +46 (0)70-497 3707

The Data Protection Officer can be contacted at: [dataskydd@rkh.se](mailto:dataskydd@rkh.se).

If you are dissatisfied with the way your personal data are processed, you have the right to lodge a complaint with the Swedish Authority for Privacy Protection (Integritetsskyddsmyndigheten), which is the supervisory authority.

**Do I have to participate in the project?**

Participation is entirely voluntary, and you may withdraw from the study at any time without providing a reason. Choosing not to participate, or deciding to withdraw, will not affect you in any way.

What happens if something goes wrong?

No risks associated with participation in this project have been identified. If you choose to participate, you will receive written information including contact details for those responsible for the project.

Are there any benefits to participating?

No financial compensation will be provided. Participation will not result in any direct personal benefit to you. However, we hope that the findings of this study may contribute to the development of a competency framework for specialist nurses in infectious diseases nursing.

If you would like further information regarding this study, please contact:

Johannes Haid
Registered Nurse, Specialist Nurse in Infectious Diseases Nursing
Master’s Student
Umeå University
Email: [johannes.haid@live.se](mailto:johannes.haid@live.se)
Mobile: +46 (0)70-774 4108

Jason Murphy
Senior Lecturer, PhD, MMSPH, Registered Nurse
Specialist Nurse in Infectious Diseases Nursing
Principal Investigator
Swedish Red Cross University
Email: [jason.murphy@rkh.se](mailto:jason.murphy@rkh.se)
Telephone: +46 (0)8-587 516 00
Mobile: +46 (0)70-497 3707

For contact with the Data Protection Officer, please email:
[dataskydd@rkh.se](mailto:dataskydd@rkh.se).
