# Supplementary material for: Identification of required competencies for specialist nurses in infectious disease care: A modified Delphi study
Source: Int J Nurs Stud Adv. 2026 May 27;11:100573. doi: 10.1016/j.ijnsa.2026.100573 (PMC13266131; doi:10.1016/j.ijnsa.2026.100573)
Supplement: Supplementary file 2 [file mmc2.docx]

# Appendices and Information Letter (Translated to American Academic English)

## Appendix 2. Questionnaire Delphi Round 1

### Section 1 of 4

Consensus Study on Key Competencies for Specialist Nurses in Infectious Disease Care: Delphi Round 1

Follow the link for the information letter to research participants.

Email:

_________________________________________________________

This form collects email addresses.

### Consent to Participate in the Project

I have received oral and/or written information about the study and have had the opportunity to ask questions. I may keep the written information.

☐ I consent to participate in the project: Consensus Study on Key Competencies for Specialist Nurses in Infectious Disease Care.

### Section 2 of 4

#### Demographic Questions

• Please state your age in years:

• Please state the number of years of professional experience in infectious disease care:

• Do you currently work clinically in infectious disease care? If not, how long has it been since you last worked clinically in infectious disease care?

- ☐ Yes

- ☐ No, less than 6 months ago

- ☐ No, 6–12 months ago

- ☐ No, 1–2 years ago

- ☐ No, 2–5 years ago

- ☐ No, 5–10 years ago

- ☐ No, more than 10 years ago

• Are you a specialist nurse in infectious disease care?

- ☐ Yes

- ☐ No

• Are you a specialist nurse in another field? If yes, please specify:

• What is your highest academic degree in nursing science?

- ☐ Bachelor’s degree

- ☐ Postgraduate Diploma

- ☐ Master’s degree

- ☐ Licentiate degree

- ☐ Doctoral degree

• Are you currently enrolled in doctoral studies?

- ☐ Yes

- ☐ No

### Section 3 of 4

#### Competencies

Please indicate how important you consider each competency for a specialist nurse in infectious disease care on a scale from 1 (not important) to 5 (very important).

• In-depth knowledge of the most common infectious diseases regarding etiology, physiology, pathophysiology, and treatment.

• In-depth knowledge of neglected tropical infectious diseases regarding etiology, physiology, pathophysiology, and treatment.

• In-depth knowledge of microbiology.

• In-depth knowledge of immunology.

• Ability to work toward reducing antibiotic resistance through advanced knowledge of antibiotics.

• Knowledge of caring for patients exposed to violence and basic understanding of the relationship between violence and health.

• Increased understanding of sustainable development, considering ethical aspects, gender equality, and human rights.

• Advanced knowledge and ability to promote sustainable decisions within the organization.

• Advanced skills in assessing and analyzing nursing needs and justifying nursing interventions related to isolation care.

• Increased understanding of and ability to promote person-centered activities and processes in infectious disease care.

• In-depth knowledge of care and nursing from a transcultural perspective.

• In-depth knowledge and understanding of donning and doffing appropriate protective equipment for various infectious diseases.

• Ability to assess, propose, and justify infection prevention measures.

• Fundamental knowledge of the Communicable Diseases Act.

• Basic knowledge of infection control measures for highly contagious and serious diseases.

• Knowledge and understanding of guidelines for working with highly contagious and serious diseases.

• Fundamental knowledge of symptoms associated with highly contagious and serious diseases.

• Ability to propose and justify pharmacological treatment related to infectious diseases.

• Ability to critically review and evaluate medication prescriptions for infectious diseases.

• Knowledge and understanding of empirical antibiotic selection for adults in hospital settings.

• Knowledge and understanding of empirical antibiotic selection for adults in outpatient care.

• Advanced skills in assessing and analyzing nursing problems and justifying nursing interventions for various types of infectious diseases.

• In-depth knowledge of preventive nursing interventions for patients with pneumonia.

• In-depth knowledge of preventive nursing interventions for patients with CNS infection.

• In-depth knowledge of preventive nursing interventions for patients with spondylodiscitis.

• In-depth knowledge of preventive nursing interventions for patients with endocarditis.

• In-depth knowledge of preventive nursing interventions for patients with tuberculosis.

• In-depth knowledge of preventive nursing interventions for patients undergoing long-term antibiotic treatment.

• Ability to systematically conduct and evaluate physical health assessments with an ethical and equitable approach.

• Ability to systematically conduct and evaluate mental health assessments with an ethical and equitable approach.

• Good clinical situational awareness to prevent critical situations for patients.

• Ability to promptly detect, assess, and analyze symptoms of necrotizing fasciitis.

• Ability to promptly detect, assess, and analyze symptoms of acute bacterial meningitis.

• Ability to promptly detect, assess, and analyze symptoms of septic shock.

• In-depth knowledge of various types of vascular access devices and related complications to minimize the risk of healthcare-associated injuries.

• Ability to assess the need for, propose, and justify the initiation of central venous access.

• Ability to conduct systematic literature reviews and apply evidence-based guidelines within the professional organization.

• In-depth knowledge of risk prevention work in the organization.

• Advanced pedagogical knowledge and ability to stimulate experiential learning among nursing students.

• Advanced pedagogical knowledge and ability to supervise undergraduate students.

• Advanced pedagogical knowledge and ability to supervise graduate students.

• Advanced pedagogical knowledge and ability to contribute to education within the clinical setting.

• Advanced pedagogical knowledge and ability to participate in external educational initiatives.

• Ability to guide colleagues to increase situational awareness and prevent critical situations for patients.

• In-depth knowledge of and ability to actively improve psychosocial health for individuals living with or diagnosed with chronic infectious diseases.

• Ability to identify and relate determinants of health for vulnerable and marginalized groups in infectious disease care, as well as to plan, design, and follow up on relevant nursing interventions.

• Epidemiological knowledge of infectious diseases at the national level.

• Epidemiological knowledge of infectious diseases at the global level.

• Basic knowledge of the national vaccination program.

• In-depth knowledge of and ability to stay updated on microbiological sampling and specimen handling.

• Ability to evaluate health-promoting and disease-preventive measures from ethical, societal, and scientific perspectives, with special consideration for human rights.

• Ability and proficiency to search for, evaluate, and implement nursing research and theories of care.

• Ability to initiate and participate in research and development activities based on a scientific approach.

• Ability to promote an evidence-based knowledge culture in the workplace.

### Section 4 of 4

Questions 55–59. Your Suggestions for Competencies

Do you think there are competencies not represented above? If yes, please describe them below.
